# The Yale Food Addiction Scale 2.0 and the modified Yale Food Addiction Scale 2.0 in Taiwan: Factor structure and concurrent validity

**DOI:** 10.3389/fpsyt.2022.1014447

**Published:** 2022-11-25

**Authors:** I-Hua Chen, Po-Ching Huang, Yi-Ching Lin, Wan Ying Gan, Chia-Wei Fan, Wen-Chi Yang, Serene En Hui Tung, Wai Chuen Poon, Mark D. Griffiths, Chung-Ying Lin

**Affiliations:** ^1^Chinese Academy of Education Big Data, Qufu Normal University, Qufu, China; ^2^Institute of Allied Health Sciences, College of Medicine, National Cheng Kung University, Tainan, Taiwan; ^3^Department of Early Childhood and Family Education, National Taipei University of Education, Taipei, Taiwan; ^4^Department of Nutrition, Faculty of Medicine and Health Sciences, Universiti Putra Malaysia, Selangor, Malaysia; ^5^Department of Occupational Therapy, AdventHealth University, Orlando, FL, United States; ^6^Division of Hematology and Medical Oncology, Department of Internal Medicine, E-DA Hospital, Kaohsiung, Taiwan; ^7^Faculty of School of Medicine, College of Medicine, I-Shou University, Kaohsiung, Taiwan; ^8^Infinite Power Ltd., Co., Kaohsiung, Taiwan; ^9^Division of Nutrition and Dietetics, School of Health Sciences, International Medical University, Kuala Lumpur, Malaysia; ^10^Sunway University Business School, Sunway University, Selangor, Malaysia; ^11^International Gaming Research Unit, Department of Psychology, Nottingham Trent University, Nottingham, United Kingdom; ^12^Department of Occupational Therapy, College of Medicine, National Cheng Kung University, Tainan, Taiwan; ^13^Department of Public Health, National Cheng Kung University Hospital, College of Medicine, National Cheng Kung University, Tainan, Taiwan; ^14^Biostatistics Consulting Center, National Cheng Kung University Hospital, College of Medicine, National Cheng Kung University, Tainan, Taiwan

**Keywords:** confirmatory factor analysis, food addiction, Yale Food Addiction Scale, reliability, validity, weight stigma, university students

## Abstract

**Introduction:**

The most widely used instruments to assess food addiction – the Yale Food Addiction Scale 2.0 (YFAS 2.0) and its modified version (mYFAS 2.0) – have not been validated in a Taiwanese population. The present study compared the psychometric properties between the Taiwan versions of YFAS 2.0 and mYFAS 2.0 among university students.

**Methods:**

An online survey comprising the YFAS 2.0, mYFAS 2.0, Weight Self-Stigma Questionnaire (WSSQ) and International Physical Activity Questionnaire-Short Form (IPAQ-SF) were used to assess food addiction, self-stigma, and physical activity.

**Results:**

All participants (*n* = 687; mean age = 24.00 years [SD ± 4.48 years]; 407 females [59.2%]) completed the entire survey at baseline and then completed the YFAS 2.0 and mYFAS 2.0 again three months later. The results of confirmatory factor analysis (CFA) indicated that the YFAS 2.0 and mYFAS 2.0 both shared a similar single-factor solution. In addition, both the YFAS 2.0 and mYFAS 2.0 reported good internal consistency (Cronbach’s α = 0.90 and 0.89), good test-retest reliability (ICC = 0.71 and 0.69), and good concurrent validity with the total scores being strongly associated with the WSSQ (*r* = 0.54 and 0.57; *p* < 0.01), and less strongly associated with BMI (*r* = 0.17 and 0.13; *p* < 0.01) and IPAQ-SF (*r* = 0.23 and 0.25; *p* < 0.01).

**Discussion:**

Based on the findings, the Taiwan versions of the YFAS 2.0 and mYFAS 2.0 appear to be valid and reliable instruments assessing food addiction.

## Introduction

Broadly speaking, the definition of food addiction refers to individuals’ uncontrollable desire for food intake (1). The concept of “Addiction and Related Disorders” proposed in the fifth edition of the *Diagnostic and Statistical Manual for Mental Disorders* (DSM-5) has allowed the potential diagnosis of varied behavioral addictions (2) and research investigating their potential associations has increased rapidly (1, 3). Food addiction has been shown to correlate with eating disorders such as binge eating and mood disorders such as impulsivity (1, 3, 4). In addition, food consumption is reported to alter the brain’s reward system (4), and individuals tend to increase their food intake as a compensatory behavior (typically to cope with negative situations in their lives), often resulting in weight gain (4). Consequently, these negative psychological and physiological impacts mean that food addiction is an issue that needs to be investigated.

Although food addiction is an important issue and worthy of further investigation, there have been debates in the literature in relation to the lack of appropriate instruments (5). However, since the development of the Yale Food Addiction Scale (YFAS), substantial empirical evidence showing the clinical relevance of food addiction has been documented (6–8). Therefore, food addiction is now a recognizable concept and there is growing interest among experts in the field of eating and weight disorders (5). The YFAS adapted the substance use criteria listed in the fourth edition of the *Diagnostic and Statistical Manual of Mental Disorders* (DSM-IV) (9) to quantify the presence or absence of food addiction. Prior psychometric evidence has illustrated the soundness and robustness of the YFAS in assessing food addiction (6–8, 10). Moreover, a revised second version (i.e., YFAS version 2.0; YFAS 2.0) has been developed to correspond to the latest edition of DSM (i.e., DSM-5) (11). Therefore, the diagnostic ability of the YFAS and YFAS 2.0 is clinically relevant.

The YFAS 2.0 has also been validated across different language versions with satisfactory findings (11–17). In the YFAS 2.0, the food addiction criteria comprise 12 symptoms, with the first 11 being used to assess the presence or absence of food addiction based on the DSM-5 and a twelfth symptom relating to significant impairment as a result of eating (11). The 12 symptoms are (i) substance consumption (i.e., consuming more food than intended); (ii) persistent desire (i.e., being unable to cut down or stop consuming food); (iii) time expenditure (i.e., time spent consuming food); (iv) activity reduction (i.e., giving up important activities due to consuming food); (v) knowledge consequences (i.e., keeps consuming food despite knowing physical/emotional consequences); (vi) tolerance (i.e., increase of food consumption over time); (vii) withdrawal (i.e., having withdrawal symptoms if unable to eat desired foods); (viii) social problems (i.e., consuming food despite having social or interpersonal problems); (ix) failure to fulfill role (i.e., unable to satisfy role obligations due to consuming food); (x) hazardous situations (i.e., consuming food that causes physically hazardous situations); (xi) craving (i.e., having strong desire to consume desired food); and (xii) significant impairment (i.e., having significant distress or impairments due to food consumption) (11). However, the YFAS 2.0 contains 35 items, which in the present authors’ view may cause survey fatigue at a time when healthcare providers need to quickly obtain food addiction information in a busy clinical setting. Indeed, the developers of YFAS 2.0 shared a similar opinion that the 35-item YFAS 2.0 may cause survey burden for large-scale epidemical research (18). Therefore, a shorter modified version of the YFAS 2.0 has been developed (i.e., mYFAS 2.0) (18).

The primary benefit of the mYFAS 2.0 is that it contains only 13 items with the same structure of the YFAS 2.0 (i.e., having 12 symptoms with 11 being diagnostic criteria corresponding to the criteria listed in the DSM-5 for substance use disorder) (18). The construct validity of the mYFAS 2.0 has been confirmed in prior research and has supported the 11-factor structure of the mYFAS 2.0 (18). Moreover, the association between the mYFAS 2.0 and its original version (i.e., YFAS 2.0) has been found to be satisfactory (18, 19). As far as the present authors’ are aware, the mYFAS 2.0 has been psychometrically evaluated in eight previous studies. These include the original validation study (18), as well as validations among a US population with heterogeneous ethnic groups (20), Chinese university students (21, 22), a French-speaking clinical sample (19), a Brazilian general population [Portuguese mYFAS 2.0; (23)], a non-clinical Czech sample [Czech mYFAS 2.0; (24)], and a non-clinical Italian sample [Italian mYFAS 2.0; (25)]. Given the increasing psychometric evidence of the mYFAS 2.0, healthcare providers may consider using mYFAS 2.0 in busy clinical settings.

However, among the studies evaluating the psychometric properties of the YFAS 2.0 and mYFAS 2.0, only two studies have directly compared the psychometric properties of the YFAS 2.0 and mYFAS 2.0 (18, 19). In the first study, Schulte and Gearhardt (18) compared the YFAS 2.0 and mYFAS 2.0 because their study aim was to develop a short version of YFAS 2.0. Therefore, the psychometric evidence comparing the YFAS 2.0 and mYFAS 2.0 was exploratory. In the second study, Brunault et al. (19) compared the YFAS 2.0 and mYFAS 2.0 among clinical and non-clinical samples. However, additional psychometric evidence is needed to corroborate whether these findings can be replicated in another ethnic population. More specifically, the samples in the study by Brunault et al. (19) comprised Western individuals. Therefore, verification of their findings in an Eastern sample (e.g., a Taiwanese sample) is needed.

The YFAS 2.0 and mYFAS 2.0 have both been recently validated in Chinese populations (21, 22, 26). However, the Chinese YFAS 2.0 and mYFAS 2.0 are written in simplified Chinese characters for individuals living in mainland China. To the best of the present authors’ knowledge, neither the YFAS 2.0 and the mYFAS 2.0 has been validated among Taiwanese people, who also speak Chinese but use a different written system (i.e., traditional Chinese characters) from those in mainland China (27). Therefore, it is important to examine the psychometric properties of the YFAS 2.0 and mYFAS 2.0 using traditional Chinese characters among Taiwanese individuals. This would enable healthcare providers in Taiwan to use validated versions of the YFAS 2.0 and mYFAS 2.0 to assess food addiction among Taiwanese populations.

Therefore, the present study evaluated and compared the psychometric properties of the Chinese YFAS 2.0 comprising traditional Chinese characters for the Taiwanese population (i.e., Taiwan versions of YFAS 2.0 and mYFAS 2.0). More specifically, construct validity, concurrent validity, internal consistency, and test-retest reliability of the Taiwanese versions of YFAS 2.0 and mYFAS 2.0 were examined among university students aged 20 years and older among males and females in the present study. Moreover, the present study hypothesized that the (i) Taiwan version of YFAS 2.0 would have a first-order unidimensional structure using 11 symptom scores converted from YFAS 2.0 35 items; (ii) Taiwan version of YFAS 2.0 would have a second-order factor structure using YFAS 2.0 35 item scores; and (iii) mYFAS 2.0 would have a first-order unidimensional structure using 11 symptom scores converted from 13 mYFAS 2.0 items.

## Materials and methods

### Participants and process

The present study recruited university students who met the following inclusion criteria: (i) being a student registered in a university program; (ii) being able to understand traditional Chinese characters and read them in the online survey; and (iii) being aged 20 years or older. The present study did not recruit participants aged between 18 and 20 years because the current Civil Law in Taiwan defines an adult as being aged 20 years or above (28, 29). Therefore, participants aged between 18 and 20 years needed to have their parents’ consent to participate in the present study. However, because the present study used an online survey with the adoption of e-consent, it was difficult to identify if parents provided consent for their 18–20-year-old children to participate. The corresponding author passed on the survey information to university students and university faculty members to seek their help in disseminating the survey information (i.e., utilizing a snowballing sampling method). More specifically, the survey was distributed to five universities (comprising National Cheng Kung University located in Southern Taiwan, Kaohsiung Medical University located in Southern Taiwan, Asian University located in Central Taiwan, National Taipei University of Education located in Northern Taiwan, and National Taitung University located in Eastern Taiwan). The survey was completed using *Google Forms* and all the survey items were mandatory. Moreover, the expected sample size for the present study was 500, a sample size that could satisfy the requirement of confirmatory factor analysis (CFA) in calculating precise estimations (30).

All individuals were requested to provide informed consent by clicking an ‘*agree*‘ icon after reading the survey information and confirming they would like to continue participating in the study. If an individual clicked the ‘*disagree’* icon after reading the information, the window automatically shut down and no survey questions were accessible to them. Participants obtained a 100 New Taiwan Dollar coupon (about $3.3 US) after completing the survey and providing their contact information. The total sample size was 687 participants (407 females). Three months later, the authors sent a second survey for the participants again to complete. The present study protocol was approved by the Institutional Review Board in the Chi Mei Medical Center (IRB Serial No.: 11007-006) and the Human Research Ethics Committee in the National Cheng Kung University (Approval No.: NCKU HREC-E-109-551-2).

### Measures

#### Yale Food Addiction Scale 2.0 (YFAS 2.0) and modified YFAS 2.0 (mYFAS 2.0)

The YFAS 2.0 contains 35 items rated using the following responses: never (score 0), less than monthly (score 1), once a month (score 2), 2–3 times a month (score 3), once a week (score 4), 2–3 times a week (score 5), 4–6 times a week (score 6), and every day (score 7). A sample item is “*I ate to the point where I felt physically ill*” (10, 11). The 35 items correspond to the 11 DSM-5 diagnostic criteria for substance use disorder (31) plus the symptom of impairment due to eating, and uses a similar structure to the first version of Yale Food Addiction Scale (i.e., YFAS 1.0) (9, 32). The diagnostic criteria are viewed as symptoms in food addiction (see Introduction for the specific criteria). A specific scoring method described by Gearhardt et al. (11) was used to convert the 35 items into the symptoms on a 0–1 dichotomous scale (0 indicates *non-endorsed*; 1 indicates *endorsed*).

The 13-item mYFAS 2.0 is a shorter version of the YFAS 2.0. However, the 12 symptoms remained in the mYFAS 2.0. Among the 13 items in the mYFAS 2.0, 11 items were used to assess the 11 DSM-5 diagnostic criteria and the remaining two items were used to assess significant impairment (i.e., having significant distress or impairments due to food consumption). The mYFAS 2.0 has also shown satisfactory psychometric properties (18, 24). The present study used Chinese versions of YFAS 2.0 and mYFAS 2.0 that were translated by a psychologist who is a native Chinese speaker with fluency in English.

#### Weight Self-Stigma Questionnaire (WSSQ)

The Weight Self-Stigma Questionnaire (WSSQ) contains 12 items rated on a five-point Likert scale (1 = strongly disagree; 5 = strongly agree) with a higher score indicating higher level of weight-related self-stigma. A sample item in the WSSQ is “*I caused my weight problems*” (33–35). The WSSQ used in the present study was a Chinese version which has reported promising psychometric properties in prior research (36). In the present study, the McDonald’s ω of the WSSQ was 0.95.

#### International Physical Activity Questionnaire—Short Form (IPAQ-SF)

The International Physical Activity Questionnaire—Short From (IPAQ-SF) contains seven items assessing how an individual engages in physical activity during a one-week period. A sample item in the IPAQ-SF is “*During the last 7 days, on how many days did you do vigorous physical activities?*” Individuals are then asked to respond how many hours and minutes they engaged in such physical activity. Some examples were given for the individual to evaluate what activities belong to such physical activity (e.g., swimming). Following this, the metabolic equivalent of task (MET) is calculated according to answers on the IPAQ-SF. The IPAQ-SF defines that sitting equates to 1 MET; walking to 3.3 METs, moderate physical activity to 4 METs, and vigorous physical activity to 8 METs. According to these definitions, the METs an individual engages in during 1 week can be calculated. The IPAQ-SF used in the present study was a Chinese version which has reported promising psychometric properties in prior research (37, 38). In the present study, the McDonald’s ω of the IPAQ-SF was 0.70.

#### Background information

The background information sheet contains several items asking the participants’ height (in cm), weight (in kg), sex (male or female), study program (undergraduate or postgraduate), smoking status (current smoker or non-smoker), alcohol use (alcohol user or non-user), chronic disease condition (yes or no), and age (in years). For chronic diseases, an item asked “Do you have any chronic disease?” For those answered ‘yes,’ a further question asked them to name the chronic disease(s). The participants’ height and weight were further used to calculate body mass index (BMI) with the unit of kg/m^2^.

### Data analysis

**FIGURE 1:**
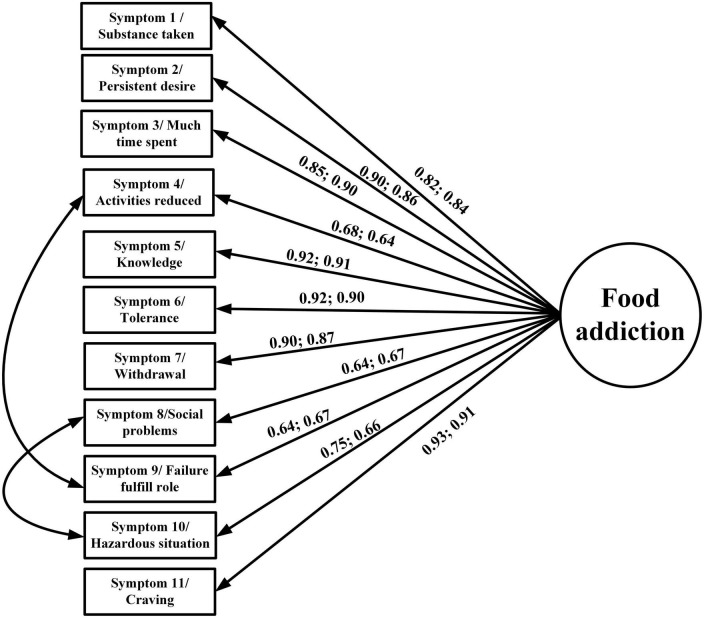
Confirmatory factor analysis (CFA) to examine the first-order structure of the YFAS 2.0 and mYFAS 2.0. Data are presented as factor loadings (aka standardized coefficients) for each YFAS 2.0 and mYFAS 2.0 symptom item, which were all stronger than 0.6.

**FIGURE 2:**
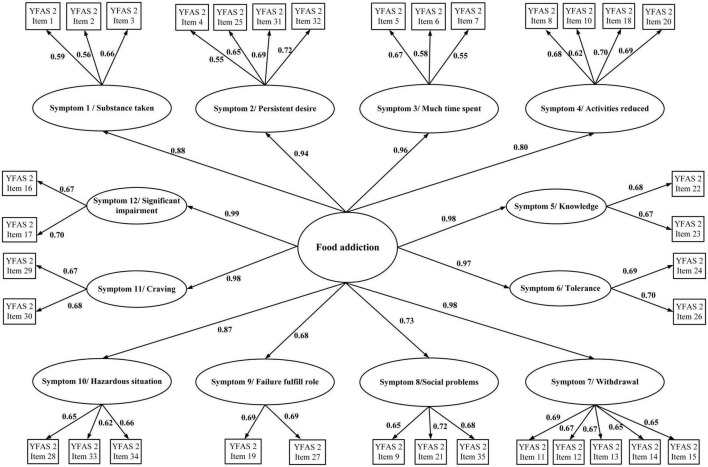
Confirmatory factor analysis (CFA) to examine the second-order structure of the YFAS 2.0. Data are presented as factor loadings (aka standardized coefficients).

In addition to the descriptive statistics, the present study used confirmatory factor analysis (CFA) to examine the factor structures of the YFAS 2.0 and mYFAS 2.0. The YFAS 2.0 was examined for two factor structures (a first-order structure and a second-order structure) according to the factor structures proposed by Manzoni et al. (39). For the first-order structure, each symptom derived from the YFAS 2.0 item calculation (please see *Measures* section for detailed scoring method) was treated as an observed score (using dichotomous scale; yes vs. no), and the first-order structure contains 11 observed item scores (Figure 1). The 12 symptoms were not included in the first-order structure because only the first 11 symptoms correspond to the DSM-5 diagnostic criteria (11). For the second-order structure, the 12 symptoms were all used as this can examine the entire YFAS 2.0. The second-order included the 12 symptoms and each symptom was constructed using 2–4 YFAS 2.0 items with a converted 0–1 scale score (please see “*Measures”* section for details). The two-factor structure is presented in Figure 2. Regarding the first-order structure for mYFAS 2.0, it is the same structure as the YFAS 2.0 first-order structure. However, the differences were mYFAS 2.0 only used one item score to indicate each symptom whereas the YFAS 2.0 used two to five item scores to indicate each symptom (also see “*Measures”* section for details).

All the CFA models were examined using maximum likelihood (ML) estimator with the asymptotic covariance matrix (ACM) and polychoric correlation (PC) because ACM and PC provide more robust and accurate estimates in ordinal scales (40). Several fit indices were used to examine the fit between the data and model: comparative fit index (CFI) > 0.95; non-normed fit index (NNFI) > 0.95; and root mean square residual of approximation (RMSEA) < 0.08 were used to indicate satisfactory fit (22). However, if the original factor structure does not have good fit indices (in any of the fit indices), a modification index was used to improve the fit. The modification index was performed one at a time and stopped when the fit became satisfactory.

After confirming the factor structure of the YFAS 2.0 and mYFAS 2.0, other psychometric properties of the YFAS 2.0 and mYFAS 2.0 were examined. More specifically, the frequency distribution of the YFAS 2.0 and mYFAS 2.0 scores were calculated for the entire sample, and then compared between sexes; multigroup CFA was conducted to examine the measurement invariance of the YFAS 2.0 and mYFAS 2.0 across sex. In the multigroup CFA, three nested models were compared: configural model, model with factor loadings constrained as equal between sex groups, and model with factor loadings and item thresholds constrained as equal between sex groups. When CFI difference (ΔCFI) > –0.01, the invariance is supported. Moreover, internal consistency was tested using McDonald’s ω; test–retest reliability using intraclass correlation coefficient (ICC); and concurrent validity (with WSSQ, BMI, and IPAQ-SF) using Pearson correlation coefficients. Moreover, ω > 0.7 and ICC > 0.4 indicate good internal consistency and test-retest reliability, respectively (22). Pearson correlation > 0.1 indicates a small effect, >0.3 indicates a moderate effect, and >0.5 indicates a strong effect for concurrent validity (41). All the statistical analyses were performed using SPSS 25.0 (IBM Corp.: Armonk, NY, USA) or LISREL 8.8 (Scientific Software: Chicago, IL, USA).

## Results

The mean age of the 687 participants was 24.0 (*SD* = 4.48) years. Moreover, there were more female participants in the present sample (*n* = 407; 59.2%). Most of the participants were studying in an undergraduate program (*n* = 489; 71.2%), a non-smoker (*n* = 663; 96.5%), a non-alcohol user (*n* = 617; 89.8%), and had no chronic disease (*n* = 649; 94.5%). The mean BMI of the participants was 22.52 kg/m^2^ (*SD* = 3.59) (Table 1). Moreover, the prevalence rate of food addiction in both the YFAS 2.0 and mYFAS 2.0 was 6.4% (*n* = 44).

**TABLE 1:** Demographic information of the participants (*N* = 687).

Age in years; Mean (*SD*)	24.00 (4.48)
Sex (Female); *n* (%)	407 (59.2%)
Academic level (Undergraduate); *n* (%)	489 (71.2%)
Smoking status (No); *n* (%)	663 (96.5%)
Alcohol status (No); *n* (%)	617 (89.8%)
Chorionic disease (No); *n* (%)	649 (94.5%)
Height in cm; Mean (*SD*)	165.54 (7.85)
Weight in kg; Mean (*SD*)	62.08 (12.73)
BMI; Mean (*SD*)	22.52 (3.59)

The original factor structures (both first-order and second-order) of the YFAS 2.0 were somewhat unsatisfactory in the RMSEA index (0.113 for first-order structure and 0.084 for second-order structure). A similarly unsatisfactory RMSEA index was found in the first-order structure of mYFAS 2.0 (0.107). After correlating two pairs of measurement errors (activities reduced with failure fulfill role; and social problems with hazardous situations), all fit indices were satisfactory for the first-order structure (For YFAS 2.0: RMSEA = 0.059, CFI = 0.995, and NNFI = 0.993; for mYFAS 2.0: RMSEA = 0.054, CFI = 0.995, and NNFI = 0.994) (Table 2). Moreover, all factor loadings of each YFAS 2.0 symptom item and each mYFAS 2.0 symptom item were stronger than 0.6 (Figure 1). After correlating two pairs of residual errors (activities reduced with failure fulfill role; and social problems with hazardous situations), all fit indices were satisfactory for the second-order structure for YFAS 2.0 (RMSEA = 0.065, CFI = 0.972, and NNFI = 0.969). Table 3 additionally reports the coefficients, standard error (SE), and factor loadings of the final CFA results for both YFAS 2.0 and mYFAS 2.0.

**TABLE 2:** Factor structure fit indices for the Yale Food Addiction Scale 2.0 (YFAS 2.0) and modified YFAS 2.0 (mYFAS 2.0).

**Instrument**				
Factor structure	Fit indices			
**YFAS 2.0**	χ^2^ (df)	RMSEA (95% CI)	CFI	NNFI
First-order structure	430.805 (44)	0.113 (0.104–0.123)	0.979	0.974
Modified first-order structure^a^	140.77 (42)	0.059 (0.048–0.069)	0.995	0.993
Second-order structure	3193.999 (548)	0.084 (0.081–0.087)	0.953	0.949
Modified second-order structure^b^	2127.938 (546)	0.065 (0.062–0.068)	0.972	0.969
**mYFAS 2.0**				
First-order structure	392.021 (44)	0.107 (0.097–0.117)	0.981	0.976
Modified first-order structure ^a^	125.250 (42)	0.054 (0.043–0.065)	0.995	0.994

CFI, comparative fit index; NNFI, non-normed fit index; RMSEA, root mean square error of approximation. *^a^*Adding the estimation of measurement errors: activities reduced with failure fulfill role; and social problems with hazardous situation. *^b^*Adding the estimation of residual errors: activities reduced with failure fulfill role; and social problems with hazardous situations.

**TABLE 3:** Coefficients, standard error (SE), and standardized coefficients (aka factor loadings) of the final confirmatory factor analysis for the Yale Food Addiction Scale 2.0 (YFAS 2.0) and modified YFAS 2.0 (mYFAS 2.0).

	Coefficient (SE)/ Standardized coefficient (All participants)	Coefficient (SE)/ Standardized coefficient (Male)	Coefficient (SE)/ Standardized coefficient (Female)
**YFAS 2.0**			
**Modified first-order structure**			
Symptom 1	0.86 (0.03)/0.82	0.87 (0.05)/0.61	0.83 (0.05)/0.80
Symptom 2	0.94 (0.02)/0.90	0.92 (0.04)/0.65	0.93 (0.02)/0.89
Symptom 3	0.89 (0.03)/0.85	0.82 (0.08)/0.58	0.90 (0.04)/0.85
Symptom 4	0.71 (0.04)/0.68	0.72 (0.05)/0.51	0.77 (0.04)/0.74
Symptom 5	0.97 (0.01)/0.92	0.94 (0.03)/0.66	0.97 (0.01)/0.93
Symptom 6	0.96 (0.02)/0.92	0.91 (0.04)/0.65	0.98 (0.02)/0.93
Symptom 7	0.94 (0.02)/0.90	0.92 (0.03)/0.65	0.95 (0.02)/0.90
Symptom 8	0.67 (0.04)/0.64	0.76 (0.05)/0.54	0.71 (0.05)/0.67
Symptom 9	0.67 (0.04)/0.64	0.69 (0.06)/0.49	0.73 (0.04)/0.70
Symptom 10	0.78 (0.03)/0.75	0.83 (0.04)/0.59	0.79 (0.04)/0.75
Symptom 11	0.98 (0.01)/0.93	1.08 (0.02)/0.76	0.97 (0.02)/0.92
**YFAS 2.0**			
**Modified second-order structure**			
YFAS2_Item 1	1.00 (ref)/0.59	1.00 (ref)/0.58	1.00 (ref)/0.55
YFAS2_Item 2	0.96 (0.08)/0.56	1.11 (0.15)/0.64	1.04 (0.13)/0.57
YFAS2_Item 3	1.14 (0.09)/0.66	1.11 (0.15)/0.64	1.17 (0.15)/0.65
YFAS2_Item 4	1.00 (ref)/0.55	1.00 (ref)/0.56	1.00 (ref)/0.56
YFAS2_Item 5	1.17 (0.10)/0.65	1.20 (0.12)/0.68	1.14 (0.11)/0.64
YFAS2_Item 6	1.24 (0.11)/0.69	1.22 (0.14)/0.68	1.23 (0.12)/0.69
YFAS2_Item 7	1.30 (0.10)/0.72	1.30 (0.13)/0.73	1.28 (0.12)/0.72
YFAS2_Item 8	1.00 (ref)/0.67	1.00 (ref)/0.67	1.00 (ref)/0.68
YFAS2_Item 9	0.86 (0.08)/0.58	0.86 (0.10)/0.58	0.84 (0.09)/0.57
YFAS2_Item 10	0.82 (0.07)/0.55	0.83 (0.10)/0.56	0.84 (0.08)/0.57
YFAS2_Item 11	1.00 (ref)/0.68	1.00 (ref)/0.67	1.00 (ref)/0.68
YFAS2_Item 12	0.91 (0.03)/0.62	0.96 (0.04)/0.64	0.94 (0.03)/0.64
YFAS2_Item 13	1.04 (0.02)/0.70	1.06 (0.03)/0.71	1.02 (0.02)/0.69
YFAS2_Item 14	1.01 (0.02)/0.69	1.04 (0.03)/0.69	1.02 (0.02)/0.69
YFAS2_Item 15	1.00 (ref)/0.68	1.00 (ref)/0.68	1.00 (ref)/0.68
YFAS2_Item 16	1.00 (0.03)/0.67	1.04 (0.03)/0.70	1.01 (0.02)/0.68
YFAS2_Item 17	1.00 (ref)/0.69	1.00 (ref)/0.71	1.00 (ref)/0.70
YFAS2_Item 18	1.01 (0.03)/0.70	0.99 (0.03)/0.70	1.01 (0.03)/0.70
YFAS2_Item 19	1.00 (ref)/0.69	1.00 (ref)/0.68	1.00 (ref)/0.69
YFAS2_Item 20	0.98 (0.02)/0.67	0.98 (0.04)/0.67	0.98 (0.03)/0.68
YFAS2_Item 21	0.98 (0.02)/0.67	0.97 (0.03)/0.66	0.98 (0.02)/0.67
YFAS2_Item 22	0.95 (0.03)/0.65	0.97 (0.03)/0.67	0.96 (0.03)/0.66
YFAS2_Item 23	0.95 (0.04)/0.65	0.93 (0.06)/0.64	0.95 (0.04)/0.65
YFAS2_Item 24	1.00 (ref)/0.65	1.00 (ref)/0.68	1.00 (ref)/0.68
YFAS2_Item 25	1.10 (0.03)/0.72	1.06 (0.03)/0.71	1.06 (0.03)/0.72
YFAS2_Item 26	1.05 (0.03)/0.68	1.02 (0.03)/0.69	0.99 (0.03)/0.67
YFAS2_Item 27	1.00 (ref)/0.69	1.00 (ref)/0.68	1.00 (ref)/0.69
YFAS2_Item 28	0.99 (0.02)/0.69	1.04 (0.02)/0.71	1.03 (0.02)/0.71
YFAS2_Item 29	1.00 (ref)/0.65	1.00 (ref)/0.66	1.00 (ref)/0.67
YFAS2_Item 30	0.96 (0.03)/0.62	0.97 (0.04)/0.64	0.95 (0.03)/0.63
YFAS2_Item 31	1.02 (0.03)/0.66	1.03 (0.04)/0.68	1.02 (0.03)/0.68
YFAS2_Item 32	1.00 (ref)/0.67	1.00 (ref)/0.68	1.00 (ref)/0.67
YFAS2_Item 33	1.01 (0.03)/0.67	0.98 (0.04)/0.67	1.01 (0.03)/0.68
YFAS2_Item 34	1.00 (ref)/0.67	1.00 (ref)/0.64	1.00 (ref)/0.67
YFAS2_Item 35	1.05 (0.02)/0.70	1.13 (0.05)/0.72	1.07 (0.03)/0.72
**mYFAS 2.0**			
**Modified first-order structure**			
Symptom 1	0.88 (0.03)/0.84	0.88 (0.05)/0.62	0.87 (0.05)/0.83
Symptom 2	0.91 (0.03)/0.86	0.89 (0.05)/0.63	0.90 (0.04)/0.85
Symptom 3	0.94 (0.03)/0.90	0.92 (0.06)/0.65	0.95 (0.03)/0.91
Symptom 4	0.67 (0.05)/0.64	0.66 (0.07)/0.47	0.72 (0.05)/0.69
Symptom 5	0.96 (0.02)/0.91	0.92 (0.03)/0.65	0.96 (0.02)/0.92
Symptom 6	0.94 (0.03)/0.90	0.87 (0.06)/0.61	0.96 (0.03)/0.91
Symptom 7	0.92 (0.02)/0.87	0.89 (0.05)/0.63	0.91 (0.03)/0.87
Symptom 8	0.71 (0.04)/0.67	0.87 (0.04)/0.61	0.67 (0.06)/0.64
Symptom 9	0.71 (0.04)/0.67	0.85 (0.05)/0.60	0.70 (0.05)/0.67
Symptom 10	0.69 (0.04)/0.66	0.72 (0.07)/0.51	0.71 (0.05)/0.68
Symptom 11	0.95 (0.02)/0.91	1.00 (0.03)/0.71	0.96 (0.02)/0.91

Given that both the YFAS 2.0 and mYFAS 2.0 had satisfactory first-order structure, the total score using the 11 (DSM-5) symptom items was used for the following psychometric testing, including frequency distributions between males and females (Table 4), measurement invariance across sex (Table 5), internal consistency, test–retest reliability (*n* = 445 participants for the test–retest), and concurrent validity. Significant differences were found between sexes in the YFAS 2.0 and mYFAS 2.0 scores. However, measurement invariance was supported for both YFAS 2.0 and mYFAS 2.0 across sex. Therefore, comparing sex using either YFAS 2.0 or mYFAS 2.0 is deemed appropriate. The impacts of sex on YFAS 2.0 or mYFAS 2.0 scores do not appear to be serious. For internal consistency, both YFAS 2.0 and mYFAS 2.0 had McDonald’s ω greater than 0.7 (0.96 for YFAS 2.0; 0.87 for mYFAS 2.0). For test-retest reliability, both YFAS 2.0 and mYFAS 2.0 had an ICC greater than 0.4 (0.71 for YFAS 2.0; 0.69 for mYFAS 2.0). For concurrent validity, both YFAS 2.0 and mYFAS 2.0 total scores had strong associations with WSSQ (*r* = 0.54 and 0.57) and small to moderate associations with BMI (*r* = 0.17 and 0.13) and IPAQ-SF (*r* = 0.23 and 0.25). Tables 6, 7 additionally report the details regarding the associations between each symptom of YFAS 2.0 (and that of mYFAS 2.0) and external criteria of WSSQ, BMI, and IPAQ-SF.

**TABLE 4:** Frequency distributions of the Yale Food Addiction Scale 2.0 (YFAS 2.0) and modified YFAS 2.0 (mYFAS 2.0) between male and female.

*N* (%) Score	All participants (*N* = 687)	Male (*N* = 280)	Female (*N* = 407)	χ^2^ (*p*-value)
**YFAS 2.0**				
0	344 (50.1)	126 (45.0)	218 (53.6)	23.41 (0.02)
1	54 (7.9)	24 (8.6)	30 (7.4)	
2	32 (4.7)	20 (7.1)	12 (2.9)	
3	55 (8.0)	31 (11.1)	24 (5.9)	
4	107 (15.6)	44 (15.7)	63 (15.5)	
5	20 (2.9)	10 (3.6)	10 (2.5)	
6	13 (1.9)	2 (0.7)	11 (2.7)	
7	11 (1.6)	6 (2.1)	5 (1.2)	
8	10 (1.4)	5 (1.8)	5 (1.2)	
9	8 (1.1)	1 (0.4)	7 (1.7)	
10	11 (1.6)	4 (1.4)	7 (1.7)	
11	22 (3.2)	7 (2.5)	15 (3.7)	
**mYFAS 2.0**				
0	373 (54.3)	143 (51.1)	230 (56.5)	13.39 (0.26)
1	69 (10.0)	39 (13.9)	30 (7.4)	
2	42 (6.1)	16 (5.7)	26 (6.4)	
3	30 (4.4)	14 (5.0)	16 (3.9)	
4	91 (13.2)	36 (12.9)	55 (13.5)	
5	22 (3.2)	11 (3.9)	11 (2.7)	
6	10 (1.5)	3 (1.1)	7 (1.7)	
7	11 (1.6)	5 (1.8)	6 (1.5)	
8	10 (1.5)	4 (1.4)	6 (1.5)	
9	10 (1.5)	5 (1.8)	5 (1.2)	
10	5 (0.7)	1 (0.4)	4 (1.0)	
11	14 (2.0)	3 (1.1)	11 (2.7)	

**TABLE 5:** Measurement invariance of the Yale Food Addiction Scale 2.0 (YFAS 2.0) and modified YFAS 2.0 (mYFAS 2.0) across sex.

	Configural model	Loadings constrained as equal	Loadings and thresholds constrained as equal
***YFAS 2.0 Modified first-order structure***			
*X ^2^*(*df*) or Δ*X ^2^*(Δ*df*)	227.64 (84)	25.25 (10)	20.23 (10)
*CFI* or Δ*CFI*	0.997	–0.009	0.000
***YFAS 2.0 Modified second-order structure***			
*X ^2^*(*df*) or Δ*X ^2^*(Δ*df*)	232.19 (84)	418.05 (10)	24.7 (10)
*CFI* or Δ*CFI*	0.989	0.004	0.005
***mYFAS 2.0 Modified first-order structure***			
*X ^2^*(*df*) or Δ*X ^2^*(Δ*df*)	3055.61 (1092)	37.48 (35)	27.72 (23)
*CFI* or Δ*CFI*	0.966	0.000	0.001

CFI, comparative fit index, and ΔCFI > –0.01 indicates measurement invariance.

**TABLE 6:** Correlation between Yale Food Addiction Scale 2.0 (YFAS 2.0) and other variables *N* = 687.

	Mean (*SD*)/ *n* (%)	1	2	3	4	5	6	7	8	9	10	11	12	13	14	15
(1) YFAS 2.0 total score	2.14 (2.91)	1.00														
(2) Symptom/substance taken (Y)	58 (8.44%)	0.59 (<0.01)	1.00													
(3) Symptom/persistent desire (Y)	70 (10.19%)	0.67 (<0.01)	0.50 (<0.01)	1.00												
(4) Symptom/much time spent (Y)	46 (6.70%)	0.59 (<0.01)	0.55 (<0.01)	0.58 (<0.01)	1.00											
(5) Symptom/activities reduced (Y)	251 (36.54%)	0.79 (<0.01)	0.26 (<0.01)	0.26 (<0.01)	0.26 (<0.01)	1.00										
(6) Symptom/knowledge (Y)	78 (11.35%)	0.76 (<0.01)	0.55 (<0.01)	0.67 (<0.01)	0.51 (<0.01)	0.39 (<0.01)	1.00									
(7) Symptom/tolerance (Y)	52 (7.57%)	0.69 (<0.01)	0.57 (<0.01)	0.70 (<0.01)	0.56 (<0.01)	0.30 (<0.01)	0.68 (<0.01)	1.00								
(8) Symptom/withdrawal (Y)	102 (14.85%)	0.72 (<0.01)	0.49 (<0.01)	0.63 (<0.01)	0.53 (<0.01)	0.36 (<0.01)	0.69 (<0.01)	0.59 (<0.01)	1.00							
(9) Symptom/social problems (Y)	264 (38.43%)	0.77 (<0.01)	0.23 (<0.01)	0.26 (<0.01)	0.23 (<0.01)	0.84 (<0.01)	0.37 (<0.01)	0.29 (<0.01)	0.34 (<0.01)	1.00						
(10) Symptom/failure fulfill role (Y)	258 (37.56%)	0.76 (<0.01)	0.22 (<0.01)	0.23 (<0.01)	0.21 (<0.01)	0.84 (<0.01)	0.37 (<0.01)	0.30 (<0.01)	0.33 (<0.01)	0.80 (<0.01)	1.00					
(11) Symptom/hazardous situation (Y)	222 (32.31%)	0.79 (<0.01)	0.26 (<0.01)	0.35 (<0.01)	0.26 (<0.01)	0.74 (<0.01)	0.46 (<0.01)	0.34 (<0.01)	0.45 (<0.01)	0.73 (<0.01)	0.71 (<0.01)	1.00				
(12) Symptom/craving (Y)	69 (10.04%)	0.75 (<0.01)	0.56 (<0.01)	0.69 (<0.01)	0.57 (<0.01)	0.38 (<0.01)	0.71 (<0.01)	0.67 (<0.01)	0.68 (<0.01)	0.32 (<0.01)	0.36 (<0.01)	0.43 (<0.01)	1.00			
(13) WSSQ	31.10 (10.49)	0.54 (<0.01)	0.21 (<0.01)	0.27 (<0.01)	0.22 (<0.01)	0.49 (<0.01)	0.38 (<0.01)	0.29 (<0.01)	0.35 (<0.01)	0.50 (<0.01)	0.47 (<0.01)	0.53 (<0.01)	0.31 (<0.01)	1.00		
(14) BMI	22.52 (3.59)	0.17 (<0.01)	0.12 (<0.01)	**0.07 (0.07)**	0.09 (0.02)	0.17 (<0.01)	0.09 (0.02)	0.09 (0.02)	0.10 (0.01)	0.20 (<0.01)	0.18 (<0.01)	0.10 (0.01)	**0.04 (0.31)**	0.31 (<0.01)	1.00	
(15) IPAQ-SF	3237.44 (2774.57)	0.23 (<0.01)	0.08 (0.04)	**0.05 (0.17)**	0.08 (0.04)	0.25 (<0.01)	0.13 (<0.01)	0.11 (<0.01)	0.09 (0.02)	0.25 (<0.01)	0.22 (<0.01)	0.28 (<0.01)	0.11 (<0.01)	0.18 (<0.01)	**0.00 (0.91)**	1.00

All correlations were significant except for those in bold, which did not meet *p* < 0.05. WSSQ, Weight Self-Stigma Questionnaire; BMI, body mass index; IPAQ-SF, International Physical Activity Questionnaire-Short Form.

**TABLE 7:** Correlation between modified Yale Food Addiction Scale 2.0 (mYFAS 2.0) and other variables *N* = 687.

	Mean (*SD*)/ *n* (%)	1	2	3	4	5	6	7	8	9	10	11	12	13	14	15
(1) mYFAS 2.0 total score	1.79 (2.66)	1.00														
(2) Symptom/substance taken (Y)	43 (6.26%)	0.58 (<0.01)	1.00													
(3) Symptom/persistent desire (Y)	55 (8.01%)	0.61 (<0.01)	0.41 (<0.01)	1.00												
(4) Symptom/much time spent (Y)	32 (4.66%)	0.60 (<0.01)	0.63 (<0.01)	0.52 (<0.01)	1.00											
(5) Symptom/activities reduced (Y)	226 (32.89%)	0.75 (<0.01)	0.24 (<0.01)	0.23 (<0.01)	0.26 (<0.01)	1.00										
(6) Symptom/knowledge (Y)	74 (10.77%)	0.74 (<0.01)	0.55 (<0.01)	0.59 (<0.01)	0.53 (<0.01)	0.38 (<0.01)	1.00									
(7) Symptom/tolerance (Y)	40 (5.82%)	0.64 (<0.01)	0.50 (<0.01)	0.64 (<0.01)	0.54 (<0.01)	0.26 (<0.01)	0.60 (<0.01)	1.00								
(8) Symptom/withdrawal (Y)	75 (10.92%)	0.64 (<0.01)	0.49 (<0.01)	0.60 (<0.01)	0.59 (<0.01)	0.26 (<0.01)	0.60 (<0.01)	0.55 (<0.01)	1.00							
(9) Symptom/social problems (Y)	208 (30.28%)	0.80 (<0.01)	0.29 (<0.01)	0.27 (<0.01)	0.25 (<0.01)	0.72 (<0.01)	0.42 (<0.01)	0.28 (<0.01)	0.28 (<0.01)	1.00						
(10) Symptom/failure fulfill role (Y)	218 (31.73%)	0.79 (<0.01)	0.26 (<0.01)	0.23 (<0.01)	0.27 (<0.01)	0.73 (<0.01)	0.39 (<0.01)	0.30 (<0.01)	0.28 (<0.01)	0.79 (<0.01)	1.00					
(11) Symptom/hazardous situation (Y)	196 (28.53%)	0.80 (<0.01)	0.24 (<0.01)	0.24 (<0.01)	0.24 (<0.01)	0.73 (<0.01)	0.42 (<0.01)	0.30 (<0.01)	0.29 (<0.01)	0.83 (<0.01)	0.82 (<0.01)	1.00				
(12) Symptom/craving (Y)	61 (8.88%)	0.71 (<0.01)	0.53 (<0.01)	0.62 (<0.01)	0.56 (<0.01)	0.33 (<0.01)	0.67 (<0.01)	0.60 (<0.01)	0.56 (<0.01)	0.36 (<0.01)	0.36 (<0.01)	0.38 (<0.01)	1.00			
(13) WSSQ	31.10 (10.49)	0.57 (<0.01)	0.22 (<0.01)	0.28 (<0.01)	0.25 (<0.01)	0.48 (<0.01)	0.38 (<0.01)	0.27 (<0.01)	0.29 (<0.01)	0.58 (<0.01)	0.52 (<0.01)	0.55 (<0.01)	0.31 (<0.01)	1.00		
(14) BMI	22.52 (3.59)	0.13 (<0.01)	**0.06 (0.10)**	0.08 (0.04)	0.09 (0.02)	0.15 (<0.01)	**0.06 (0.14)**	**0.05 (0.23)**	0.10 (0.01)	0.14 (<0.01)	0.13 (<0.01)	0.08 (0.04)	**0.03 (0.50)**	0.31 (<0.01)	1.00	
(15) IPAQ-SF	3237.44 (2774.57)	0.25 (<0.01)	**0.07 (0.06)**	**0.02 (0.56)**	0.10 (0.01)	0.23 (<0.01)	0.10 (0.01)	0.08 (0.03)	0.06 **(0.11)**	0.29 (<0.01)	0.29 (<0.01)	0.31 (<0.01)	0.11 (<0.01)	0.18 (<0.01)	**0.00 (0.91)**	1.00

All correlations were significant except for those in bold, which did not meet *p* < 0.05. WSSQ, Weight Self-Stigma Questionnaire; BMI, body mass index; IPAQ-SF, International Physical Activity Questionnaire-Short Form.

## Discussion

The present study examined and compared the psychometric properties between the Taiwan versions of the YFAS 2.0 and the mYFAS 2.0 written in traditional Chinese characters among Taiwanese university students. The results concerning construct validity showed that both the YFAS 2.0 and the mYFAS 2.0 shared the same single-factor structural solution, which had a satisfactory fit with the construct of food addiction. In addition, the second-order construct of the Taiwan version YFAS 2.0 was further examined and the results demonstrated a well-fitting structure as supported by all fit indices. Other psychometric characteristics (including internal consistency, test–retest reliability and concurrent validity) were also satisfactory for both YFAS 2.0 and mYFAS 2.0, and suggest that the two instruments have relatively equivalent psychometric properties.

The YFAS 2.0 has been translated and validated in several language, including English (i.e., the original version) (11), Italian (12, 39), German (42), French (13), Spanish (15), Arabic (14), Turkish (43), Japanese (16), Persian (44), and Portuguese (45). The present findings showed that the Taiwan version of YFAS 2.0 tested in the present study is comparable to all other language versions. Most of the previous studies have demonstrated acceptable construct validity of a single-factor structure for the YFAS 2.0, except for a study conducted among the French population (13) which showed a somewhat inferior fit to the model. Regarding internal consistency, all previous studies showed that the YFAS 2.0 is acceptable with a good α value (11–16, 39, 42–45). Furthermore, the concurrent validity examined via the associations between food addiction and related physiological behaviors or psychological symptoms were conducted and confirmed in most studies.

In particular, BMI, which was used in the original study (11), is often targeted to validate the construct of food addiction. The significant correlation between BMI and food addiction has been reported in previous studies (11, 13, 15, 16, 42, 44, 45), including the present one. Other validated measures such as those assessing binge eating (11–13, 16, 39) or food craving (42, 44) are commonly used and are significantly correlated with scores on the YFAS 2.0. Despite the aforementioned good properties, to the best of the present authors’ knowledge, only three studies, including the present one, have reported test-retest reliability for the YFAS 2.0 (14, 39). All three studies indicated that the YFAS 2.0 had good test–retest reliability. In addition, the YFAS 2.0 has been administered among different populations for food addiction prevalence assessment. For example, some studies have targeted clinical populations (e.g., individuals with obesity, eating disorders, and other potentially addictive behaviors) and reported prevalence rates of 24–77.8% (15, 39, 42). Some have targeted populations such as university students and reported prevalence rates of 3.3–11% (12, 14, 16, 42, 45). The prevalence rate of 6.4% among university students in the present study fits within this range. Other studies have targeted the general population and reported prevalence rates of 3.3–8.2% (13, 15, 39, 44). These studies suggest that the YFAS 2.0 had a good feasibility for varied implementation.

The mYFAS 2.0 has been translated and validated in several languages, including English (i.e., the original version) (18), Italian (25), Brazilian (23), Czech (24), French (19), Chinese (21, 22), and Taiwanese (i.e., the present study). Two studies from the US (46) and Egypt (i.e., Arabic language) (47) have also reported partial psychometric characteristics for the mYFAS 2.0. Most of these studies have reported adequate to good construct validity of a single-factor structure, except for one study conducted in mainland China (21) which reported a two-factor structure. This may be due to transcultural adjustment (i.e., the cross-cultural item adjustment affects the scale’s original factor structure) and regional influences (e.g., the unique dietary habits in the area the study was carried out). Regarding the internal consistency, all previous studies have shown that the mYFAS 2.0 has a good α value (18, 19, 21–25, 47). Moreover, the concurrent validity verified through the correlations between food addiction and the selected measures have demonstrated good to excellent results in all previous studies. BMI has been the most frequently used measure to examine the concurrent validity of the mYFAS 2.0 (19, 21, 22, 47). Other measures such as impulsivity (21, 23) and binge eating (19, 22) have also been used in other studies as the validators and have found to be significantly associated with food addiction as assessed using the mYFAS 2.0. Despite of the good aforementioned psychometric characteristics, to the best of the present authors’ knowledge (and like the YFAS), there have only been three studies (21, 22), including the present one, that have reported the test-retest reliability of the mYFAS 2.0 Furthermore, mYFAS 2.0 has also been implemented on different populations. One conducted in France (19) targeted a clinically obese population and reported a prevalence rate of 20.6%. Two of them targeted university students and reported prevalence rates of 6.2 and 6.7% (21, 22) which are very similar to the prevalence rate of 6.4% among university students in the present study. Other studies targeted on general population and reported prevalence rates of 4.3–17.6% (18, 19, 23–25, 46, 47). Like the YFAS 2.0, these studies suggest that the mYFAS 2.0 had a good feasibility for varied implementation irrespective of the types of group investigated.

Both the YFAS 2.0 and the mYFAS 2.0 are supported by a single-factor structure with 11 criteria and this structure can be explained by the 11 criteria described in the DSM-5. Substance-use disorder (SUD) comprises a pattern of symptoms derived from the continued use of a substance despite experiencing problems as a result (48). The concept of food addiction has been much debated particularly because food is essential for life maintenance rather than a psychoactive substance, therefore some claim it cannot be addictive (49).

However, the concept of food addiction has been reinforced by both animal studies (50, 51) and human studies (52, 53) which have shown the addictive-like responses and the withdrawal symptoms derived from food. This is why Gearhardt et al. (9) developed the first version of YFAS, and adopted the seven criteria of substance use listed in DSM-IV. With the publication of the DSM-5, the updated version of YFAS 2.0 (11) was developed according to the 11 revised diagnostic criteria. In the DSM-5, substance use was considered as a single disorder with different severity levels (31). The new diagnostic criteria combined the original criteria from DSM-IV with the four new concepts including “craving” resulting in 11 criteria (31), which basically refer to symptoms of “loss of behavioral control” that underpin addictive behavior (48). Therefore, the items in the YFAS 2.0 modeled the symptoms of addictive behavior to provide a solid construct of food addiction.

Research evidence has supported the association between weight-related self-stigma and BMI to food addiction (54). A theoretical model of cyclic obesity/weight-based stigma (COBWEBS) model (55) proposed that weight stigma generates the stress which evokes the eating disorder and increases food intake. The consequential weight gain and obesity worsen the stigmatization toward the individuals resulting in a vicious cycle. More specifically, stigmatized individuals internalize the perceived weight stigma to form the weight-related self-stigma (56). The resulting psychological distress (56) and vulnerability (57) may evoke an eating disorder which gradually develops into food addiction (54), causing an increased BMI and obesity. Moreover, the association between obesity and physical activity level is frequently reported (58). However, the mechanism remains unclear. The extant literature supports the hypothetical theory regarding the potential contribution of the dopamine motive system (59, 60). In the animal model, chronic exposure to obesogenic food impairs dopamine regulation (61). This further affects the control of effortful movement (59) and results in weight gain by reducing the physical activity level (60, 62). This theory may explain the alteration of physical activity level among individuals with food addiction and the reported association in the present study further supports this contention.

There are some limitations in the present study. First, the sample population (i.e., university students) in the present study may mean the findings lack generalizability. More specifically, food addiction has been reported to have a higher prevalence among younger age populations (6, 18, 24). Therefore, the recruitment of university students as the present study’s population may have influenced the psychometric characteristics (e.g., construct validity or concurrent validity) in the present study. Second, the self-reported evaluation used in the present study may be subject to some study biases. For example, recall bias (e.g., the participants may have inaccurately recalled their food intake in the past 12 months), single rater bias (e.g., the participants may have had an error judgment due to their potential characteristics), and social desirability bias (e.g., the participants may have intentionally reported they had less food intake than they actually did). Third, the present study did not set questions to avoid bot respondents. However, the responses were closely checked and neither specific response pattern (e.g., answering all items in the same way) nor extreme height and weight (e.g., height over 200 cm) were found in the responses. Fourth, both Chinese version of YFAS 2.0 and mYFAS 2.0 used in the present study did not follow the international guidelines for translation. Instead, we obtained the scales from a psychologist who was approved to translate by the developers. Therefore, the linguistic validity is unknown. However, given that the present findings showed good psychometric results for both YFAS 2.0 and mYFS 20, the present authors do not believe the linguistic validity to be a serious issue. Fifth, the psychiatric status of respondents was not evaluated because the survey was conducted online. Psychiatric conditions might have impacted the respondents’ ability to answer the online survey therefore it is unknown to what extent this may have biased the present study’s findings. Nevertheless, the present study provided the information regarding the psychometric properties of the Taiwan versions of the YFAS 2.0 and the mYFAS 2.0. Both instruments had a good fit with regard to construct validity and had similarity in concurrent validity. Therefore, it can be concluded that Taiwan versions of the YFAS 2.0 and the mYFAS 2.0 appear to be valid and reliable instruments for assessing food addiction.

## Conclusion

Both the YFAS 2.0 and the mYFAS 2.0 appear to be robust instruments for assessing food addiction among Taiwanese young people. Their psychometric properties included a unidimensional structure corresponding to the DSM-5 criteria on SUD, a good test–retest reliability, and satisfactory concurrent validity. Based on the psychometric evidence, healthcare providers and food addiction researchers in Taiwan could use either the YFAS 2.0 or the mYFAS 2.0 to assess food addiction among the Taiwanese population (although further testing in non-university student populations is needed). Moreover, the mYFAS 2.0 has the advantage of being brief. Therefore, it may be more suitable than the YFAS 2.0 to be used in a busy clinical setting as a quick screening tool. In contrast, the YFAS 2.0 (as compared with the mYFAS 2.0) is perhaps more appropriate to use in the comprehensive assessment of food addiction, particularly in treatment settings.

## Data availability statement

The raw data supporting the conclusions of this article will be made available by the authors, without undue reservation.

## Ethics statement

The studies involving human participants were reviewed and approved by the Institutional Review Board in the Chi Mei Medical Center (IRB Serial No.: 11007-006) and the Human Research Ethics Committee in the National Cheng Kung University (Approval No. NCKU HREC-E-109-551-2). The patients/participants provided their written informed consent to participate in this study.

## Author contributions

I-HC, P-CH, Y-CL, W-CY, and C-YL: substantial contributions to the conception or design of the work. I-HC, P-CH, WG, C-WF, W-CY, ST, WP, MDG, and C-YL: acquisition, analysis, or interpretation of data for the work. All authors drafting the work or revising it critically for important intellectual content, agreement to be accountable for all aspects of the work in ensuring that questions related to the accuracy or integrity of any part of the work are appropriately investigated and resolved.

## References

[1] Kalon E Hong JY Tobin C Schulte T. Psychological and neurobiological correlates of food addiction. *Int Rev Neurobiol.* (2016) 129:85–110. 10.1016/bs.irn.2016.06.003 27503449 PMC5608024

[2] Albayrak O Wolfle SM Hebebrand J. Does food addiction exist? A phenomenological discussion based on the psychiatric classification of substance-related disorders and addiction. *Obes Facts.* (2012) 5:165–79. 10.1159/000338310 22647300

[3] Piccinni A Bucchi R Fini C Vanelli F Mauri M Stallone T Food addiction and psychiatric comorbidities: a review of current evidence. *Eat Weight Disord.* (2021) 26:1049–56. 10.1007/s40519-020-01021-3 32968944

[4] Stice E Spoor S Bohon C Veldhuizen MG Small DM. Relation of reward from food intake and anticipated food intake to obesity: a functional magnetic resonance imaging study. *J Abnorm Psychol.* (2008) 117:924–35. 10.1037/a0013600 19025237 PMC2681092

[5] Yekaninejad MS Badrooj N Vosoughi F Lin CY Potenza MN Pakpour AH. Prevalence of food addiction in children and adolescents: a systematic review and meta-analysis. *Obes Rev.* (2021) 22:e13183. 10.1111/obr.13183 33403795 PMC8244111

[6] Pursey KM Stanwell P Gearhardt AN Collins CE Burrows TL. The prevalence of food addiction as assessed by the Yale Food Addiction Scale: a systematic review. *Nutrients.* (2014) 6:4552–90. 10.3390/nu6104552 25338274 PMC4210934

[7] Penzenstadler L Soares C Karila L Khazaal Y. Systematic review of food addiction as measured with the Yale Food Addiction Scale: implications for the food addiction construct. *Curr Neuropharmacol.* (2019) 17:526–38. 10.2174/1570159X16666181108093520 30406740 PMC6712300

[8] Praxedes DRS Silva-Junior AE Macena ML Oliveira AD Cardoso KS Nunes LO Prevalence of food addiction determined by the Yale Food Addiction Scale and associated factors: a systematic review with meta-analysis. *Eur Eat Disord Rev.* (2022) 30:85–95. 10.1002/erv.2878 34953001

[9] Gearhardt AN Corbin WR Brownell KD. Preliminary validation of the Yale Food Addiction Scale. *Appetite.* (2009) 52:430–6. 10.1016/j.appet.2008.12.003 19121351

[10] Meule A Gearhardt AN. Ten years of the Yale Food Addiction Scale: a review of version 2.0. *Curr Addict Rep.* (2019) 6:218–28. 10.1007/s40429-019-00261-3

[11] Gearhardt AN Corbin WR Brownell KD. Development of the Yale Food Addiction Scale Version 2.0. *Psychol Addict Behav.* (2016) 30:113–21. 10.1037/adb0000136 26866783

[12] Aloi M Rania M Rodriguez Munoz RC Jimenez Murcia S Fernandez-Aranda F De Fazio P Validation of the Italian version of the Yale Food Addiction Scale 2.0 (I-YFAS 2.0) in a sample of undergraduate students. *Eat Weight Disord.* (2017) 22:527–33. 10.1007/s40519-017-0421-x 28780748

[13] Brunault P Courtois R Gearhardt AN Gaillard P Journiac K Cathelain S Validation of the French version of the DSM-5 Yale Food Addiction Scale in a nonclinical sample. *Can J Psychiatry.* (2017) 62:199–210. 10.1177/0706743716673320 28212499 PMC5317020

[14] Fawzi M Fawzi M. Validation of an Arabic version of the Yale Food Addiction Scale 2.0. *East Mediterr Health J.* (2018) 24:745–52. 10.26719/2018.24.8.745 30328605

[15] Granero R Jimenez-Murcia S Gearhardt AN Aguera Z Aymami N Gomez-Pena M Validation of the Spanish version of the Yale Food Addiction Scale 2.0 (YFAS 2.0) and Clinical correlates in a sample of eating disorder, gambling disorder, and healthy control participants. *Front Psychiatry.* (2018) 9:208. 10.3389/fpsyt.2018.00208 29887808 PMC5980980

[16] Khine MT Ota A Gearhardt AN Fujisawa A Morita M Minagawa A Validation of the Japanese version of the Yale Food Addiction Scale 2.0 (J-YFAS 2.0). *Nutrients.* (2019) 11:687. 10.3390/nu11030687 30909486 PMC6471687

[17] Linardon J Messer M. Assessment of food addiction using the Yale Food Addiction Scale 2.0 in individuals with binge-eating disorder symptomatology: factor structure, psychometric properties, and clinical significance. *Psychiatry Res.* (2019) 279:216–21. 10.1016/j.psychres.2019.03.003 30862369

[18] Schulte EM Gearhardt AN. Development of the modified Yale Food Addiction Scale Version 2.0. *Eur Eat Disord Rev.* (2017) 25:302–8. 10.1002/erv.2515 28370722

[19] Brunault P Berthoz S Gearhardt AN Gierski F Kaladjian A Bertin E The modified Yale Food Addiction Scale 2.0: validation among non-clinical and clinical French-speaking samples and comparison with the full Yale Food Addiction Scale 2.0. *Front Psychiatry.* (2020) 11:480671. 10.3389/fpsyt.2020.480671 33033480 PMC7509420

[20] Carr MM Schulte EM Saules KK Gearhardt AN. Measurement invariance of the modified Yale Food Addiction Scale 2.0 across gender and racial groups. *Assessment.* (2020) 27:356–64. 10.1177/1073191118786576 29973060

[21] Zhang H Tong T Gao Y Liang C Yu H Li S Translation of the Chinese version of the modified Yale Food Addiction Scale 2.0 and its validation among college students. *J Eat Disord.* (2021) 9:116. 10.1186/s40337-021-00471-z 34530921 PMC8444594

[22] Li S Schulte EM Cui G Li Z Cheng Z Xu H. Psychometric properties of the Chinese version of the modified Yale Food Addiction Scale version 2.0 (C-mYFAS 2.0): prevalence of food addiction and relationship with resilience and social support. *Eat Weight Disord.* (2022) 27:273–84. 10.1007/s40519-021-01174-9 33779965

[23] Nunes-Neto PR Kohler CA Schuch FB Quevedo J Solmi M Murru A Psychometric properties of the modified Yale Food Addiction Scale 2.0 in a large Brazilian sample. *Braz J Psychiatry.* (2018) 40:444–8. 10.1590/1516-4446-2017-2432 29898195 PMC6899372

[24] Pipova H Kascakova N Furstova J Tavel P. Development of the modified Yale Food Addiction Scale Version 2.0 summary version in a representative sample of Czech population. *J Eat Disord.* (2020) 8:16. 10.1186/s40337-020-00292-6 32391149 PMC7197132

[25] Imperatori C Fabbricatore M Lester D Manzoni GM Castelnuovo G Raimondi G Psychometric properties of the modified Yale Food Addiction Scale Version 2.0 in an Italian non-clinical sample. *Eat Weight Disord.* (2019) 24:37–45. 10.1007/s40519-018-0607-x 30414076

[26] Chen G Tang Z Guo G Liu X Xiao S. The Chinese version of the Yale Food Addiction Scale: an examination of its validation in a sample of female adolescents. *Eat Behav.* (2015) 18:97–102. 10.1016/j.eatbeh.2015.05.002 26026613

[27] Chang CC Lin CY Gronholm PC Wu TH. Cross-validation of two commonly used self-stigma measures, Taiwan versions of the Internalized Stigma Mental Illness Scale and Self-Stigma Scale-Short, for people with mental illness. *Assessment.* (2018) 25:777–92. 10.1177/1073191116658547 27385391

[28] Chen I-H Chang K-C Chang C-W Huang S-W Potenza MN Pakpour AH Temporal associations between problematic use of the internet and self-stigma among people with substance use disorders: a cross-lagged model across one year. *J Psychiatr Res.* (2022) 156:339–48. 10.1016/j.jpsychires.2022.10.044 36323137

[29] Chang K-C Chen H-P Huang S-W Chen J-S Potenza MN Pakpour AH Comparisons of psychological distress and self-stigma among three types of substance use disorders receiving treatment-as-usual approaches: real-world data from a nine-month longitudinal study. *Ther Adv Chronic Dis*. (2022) (in press). 10.1177/20406223221140393 PMC9723802 36483780

[30] DiStefano C Shi D Morgan GB. Collapsing categories is often more advantageous than modeling sparse data: investigations in the CFA framework. *Struct Equ Modeling.* (2021) 28:237–49. 10.1080/10705511.2020.1803073

[31] American Psychological Association. *Diagnostic and Statistical Manual of Mental Disorders.* Washington, DC: American Psychological Association (2013).

[32] Manzoni GM Rossi A Pietrabissa G Varallo G Molinari E Poggiogalle E Validation of the Italian Yale Food Addiction Scale in postgraduate university students. *Eat Weight Disord.* (2018) 23:167–76. 10.1007/s40519-018-0495-0 29532419

[33] Lillis J Luoma JB Levin ME Hayes SC. Measuring weight self-stigma: the weight self-stigma questionnaire. *Obesity.* (2010) 18:971–6. 10.1038/oby.2009.353 19834462

[34] Lin CY Imani V Cheung P Pakpour AH. Psychometric testing on two weight stigma instruments in Iran: weight self-stigma questionnaire and weight bias internalized scale. *Eat Weight Disord.* (2020) 25:889–901. 10.1007/s40519-019-00699-4 31055783

[35] Gan WY Tung SEH Kamolthip R Ghavifekr S Chirawat P Nurmala I Evaluation of two weight stigma scales in Malaysian university students: weight self-stigma questionnaire and perceived weight stigma scale. *Eat Weight Disord.* (2022) 27:2595–604. 10.1007/s40519-022-01398-3 35474190

[36] Pakpour AH Tsai MC Lin YC Strong C Latner JD Fung XCC Psychometric properties and measurement invariance of the Weight Self-Stigma Questionnaire and Weight Bias Internalization Scale in children and adolescents. *Int J Clin Health Psychol.* (2019) 19:150–9. 10.1016/j.ijchp.2019.03.001 31193103 PMC6517648

[37] Liou YM Jwo CJ Yao KG Chiang LC Huang LH. Selection of appropriate Chinese terms to represent intensity and types of physical activity terms for use in the Taiwan version of IPAQ. *J Nurs Res.* (2008) 16:252–63. 10.1097/01.jnr.0000387313.20386.0a 19061172

[38] Cheng OY Yam CLY Cheung NS Lee PLP Ngai MC Lin CY. Extended theory of planned behavior on eating and physical activity. *Am J Health Behav.* (2019) 43:569–81. 10.5993/AJHB.43.3.11 31046887

[39] Manzoni GM Rossi A Pietrabissa G Mannarini S Fabbricatore M Imperatori C Structural validity, measurement invariance, reliability and diagnostic accuracy of the Italian version of the Yale Food Addiction Scale 2.0 in patients with severe obesity and the general population. *Eat Weight Disord.* (2021) 26:345–66. 10.1007/s40519-020-00858-y 32026378

[40] Zhang G Trichtinger LA Lee D Jiang G. PolychoricRM: a computationally efficient R function for estimating polychoric correlations and their asymptotic covariance matrix. *Struct Equ Modeling.* (2022) 29:310–20. 10.1080/10705511.2021.1929996

[41] Cohen J. *Statistical Power Analysis for the Behavioral Sciences.* New York, NY: Elsevier Science (2013).

[42] Meule A Muller A Gearhardt AN Blechert J. German version of the Yale Food Addiction Scale 2.0: prevalence and correlates of ‘food addiction’ in students and obese individuals. *Appetite.* (2017) 115:54–61. 10.1016/j.appet.2016.10.003 27717658

[43] Şengüzel E Öztora S Dağdeviren HN. Internal reliability analysis of the Turkish version of the Yale Food Addiction Scale. *Eur J Fam Med.* (2018) 7:14–8. 10.1186/s41043-019-0202-4 31822299 PMC6905049

[44] Haghighinejad H Tarakemehzadeh M Jafari P Jafari M Ramzi M Hedayati A. Persian version of the Yale Food Addiction Scale 2.0: psychometric analysis and setting cutoff point for the Food Cravings Questionnaire-Trait-Reduced. *Psychiatry Investig.* (2021) 18:179–86. 10.30773/pi.2020.0198 33735551 PMC8016689

[45] Goncalves S Moreira CS Machado BC Bastos B Vieira AI. Psychometric properties and convergent and divergent validity of the Portuguese Yale Food Addiction Scale 2.0 (P-YFAS 2.0). *Eat Weight Disord.* (2022) 27:791–801. 10.1007/s40519-021-01218-0 34053016

[46] Schulte EM Gearhardt AN. Associations of food addiction in a sample recruited to be nationally representative of the United States. *Eur Eat Disord Rev.* (2018) 26:112–9. 10.1002/erv.2575 29266583

[47] Mobarak EI Eldeeb D El-Weshahi H. Reliability of an Arabic version of the Short Form Modified Yale Food Addiction Scale. *J High Inst Public Health.* (2019) 49:168–74. 10.21608/JHIPH.2019.60843

[48] McLellan AT. Substance misuse and substance use disorders: why do they matter in healthcare? *Trans Am Clin Climatol Assoc.* (2017) 128:112–30. 28790493 PMC5525418

[49] Wilcox CE editor. The food addiction concept: History, controversy, potential pitfalls, and promises. *Food Addiction, Obesity, and Disorders of Overeating.* Cham: Springer (2021). p. 69–75.

[50] Oswald KD Murdaugh DL King VL Boggiano MM. Motivation for palatable food despite consequences in an animal model of binge eating. *Int J Eat Disord.* (2011) 44:203–11. 10.1002/eat.20808 20186718 PMC2941549

[51] Robinson MJ Burghardt PR Patterson CM Nobile CW Akil H Watson SJ Individual differences in cue-induced motivation and striatal systems in rats susceptible to diet-induced obesity. *Neuropsychopharmacology.* (2015) 40:2113–23. 10.1038/npp.2015.71 25761571 PMC4613617

[52] Gearhardt AN Davis C Kuschner R Brownell KD. The addiction potential of hyperpalatable foods. *Curr Drug Abuse Rev.* (2011) 4:140–5. 10.2174/1874473711104030140 21999688

[53] Schulte EM Avena NM Gearhardt AN. Which foods may be addictive? The roles of processing, fat content, and glycemic load. *PLoS One.* (2015) 10:e0117959. 10.1371/journal.pone.0117959 25692302 PMC4334652

[54] Baldofski S Rudolph A Tigges W Herbig B Jurowich C Kaiser S Weight bias internalization, emotion dysregulation, and non-normative eating behaviors in prebariatric patients. *Int J Eat Disord.* (2016) 49:180–5. 10.1002/eat.22484 26593154

[55] Tomiyama AJ. Weight stigma is stressful. A review of evidence for the Cyclic Obesity/Weight-Based Stigma model. *Appetite.* (2014) 82:8–15. 10.1016/j.appet.2014.06.108 24997407

[56] Lin CY Tsai MC Liu CH Lin YC Hsieh YP Strong C. Psychological pathway from obesity-related stigma to depression via internalized stigma and self-esteem among adolescents in Taiwan. *Int J Environ Res Public Health.* (2019) 16:4410. 10.3390/ijerph16224410 31718003 PMC6887789

[57] Hayward LE Vartanian LR Pinkus RT. Weight stigma predicts poorer psychological well-being through internalized weight bias and maladaptive coping responses. *Obesity.* (2018) 26:755–61. 10.1002/oby.22126 29427370

[58] Baillot A Chenail S Barros Polita N Simoneau M Libourel M Nazon E Physical activity motives, barriers, and preferences in people with obesity: a systematic review. *PLoS One.* (2021) 16:e0253114. 10.1371/journal.pone.0253114 34161372 PMC8221526

[59] Schmidt L Lebreton M Clery-Melin ML Daunizeau J Pessiglione M. Neural mechanisms underlying motivation of mental versus physical effort. *PLoS Biol.* (2012) 10:e1001266. 10.1371/journal.pbio.1001266 22363208 PMC3283550

[60] Volkow ND Wise RA Baler R. The dopamine motive system: implications for drug and food addiction. *Nat Rev Neurosci.* (2017) 18:741–52. 10.1038/nrn.2017.130 29142296

[61] Davis JF Tracy AL Schurdak JD Tschop MH Lipton JW Clegg DJ Exposure to elevated levels of dietary fat attenuates psychostimulant reward and mesolimbic dopamine turnover in the rat. *Behav Neurosci.* (2008) 122:1257–63. 10.1037/a0013111 19045945 PMC2597276

[62] Kravitz AV O’Neal TJ Friend DM. Do dopaminergic impairments underlie physical inactivity in people with obesity? *Front Hum Neurosci.* (2016) 10:514. 10.3389/fnhum.2016.00514 27790107 PMC5063846

